# The Importance of Vacuolar Ion Homeostasis and Trafficking in Hyphal Development and Virulence in *Candida albicans*

**DOI:** 10.3389/fmicb.2021.779176

**Published:** 2021-12-09

**Authors:** Quanzhen Lv, Lan Yan, Yuanying Jiang

**Affiliations:** ^1^School of Pharmacy, Naval Medical University, Shanghai, China; ^2^Shanghai Tenth People’s Hospital, Tongji University School of Medicine, Shanghai, China

**Keywords:** *Candida albicans*, vacuolar proton-translocating ATPases, vacuolar protein sorting components, vacuolar Ca2^+^ channel, virulence

## Abstract

The vacuole of *Candida albicans* plays a significant role in many processes including homeostasis control, cellular trafficking, dimorphic switching, and stress tolerance. Thus, understanding the factors affecting vacuole function is important for the identification of new drug targets needed in response to the world’s increasing levels of invasive infections and the growing issue of fungal drug resistance. Past studies have shown that vacuolar proton-translocating ATPases (V-ATPases) play a central role in pH homeostasis and filamentation. Vacuolar protein sorting components (VPS) regulate V-ATPases assembly and at the same time affect hyphal development. As well, vacuolar calcium exchange systems like Yvc1 and Pmc1 maintain cytosolic calcium levels while being affected by V-ATPases function. All these proteins play a role in the virulence and pathogenesis of *C. albicans*. This review highlights the relationships among V-ATPases, VPS, and vacuolar calcium exchange proteins while summarizing their importance in *C. albicans* infections.

## Introduction

*Candida albicans* is an opportunistic fungal pathogen generating a high rate of mortality in systemic infections (Jenks et al., 2020). Due to *C. albicans*’ growing resistance to antifungal drugs, there is a great need to further study the pathways affecting its pathogenesis and virulence in order to discover new potential drug targets (Berman and Krysan, 2020). Vacuoles occupy 10–20% of the yeast cell’s volume and are involved in several cellular functions including ion homeostasis, stress response, cell differentiation, and adaptation to new environments (Armstrong, 2010). Thus, vacuolar function changes can have profound effects on the virulence of *C. albicans*, and targeting the vacuolar function of *C. albicans* may provide a new strategy for the development of antifungal drugs (Olsen, 2014).

## *C. albicans* V-ATPases’ Disruption Impairs Vacuolar Acidification and Virulence

pH is a key consideration for pathogenic yeasts like *C. albicans* as it affects their virulence and dimorphic switching. pH homeostasis is not only required for sensing and responding to ambient pH, but also generating and transducing signals for secreting virulence factors (Patenaude et al., 2013; Du and Huang, 2016). The vacuolar pH is especially important for pathogenesis because vacuoles play a key role in cellular trafficking, and the defects in endosomal trafficking can affect the expression of adhesion and invasion membrane proteins (Kulkarny et al., 2014; Kim et al., 2019).

Maintaining vacuolar pH through acidification is the major role of the proton pumps called vacuolar proton-translocating ATPases (V-ATPases), which transport H^+^ from the cytoplasm into the vacuole (Parra et al., 2014). They contain a peripheral membrane subcomplex V_1_ and an integral membrane subcomplex V_0_ (Kane, 2007). The subcomplex V_1_ consists of subunits A, B, C, D, E, F, G, H, which are encoded by the genes *TFP1*, *VMA2*, *VMA5*, *VMA8*, *VMA4*, *VMA7*, *VMA10*, and *VMA13*, respectively. The subcomplex V_0_ includes subunits a, c, c’, c,” d, e, which are encoded by genes *VPH1*/*STV1*, *VMA3, VMA11*, *VMA16*, *VMA6*, and *C1_10750C_A* (Table 1). For both the non-pathogenic model yeast *S. cerevisiae* and the pathogenic *C. albicans*, all subunits are encoded by single genes, except for the subunit a in V_0_ which is encoded by the paralogs *VPH1* and *STV1* (Patenaude et al., 2013). The phenotypes of each subunit disruption mutant are summarized in Table 1.

**Table 1:** Genes encoding the subunits of V-ATPases and their null mutant phenotypes in *C. albicans*.

Subcomplexes of V-ATPase	Subunits	Encoding genes	Phenotypes of null mutant			References
			Vacuolar acidification	Hypal development	Virulence in systematic infection	
V_1_	A	*TFP1*	Decreased	Locked in yeast	Avirulent	Jia et al., 2014
	B	*VMA2*	Decreased	Locked in yeast	Avirulent	Rane et al., 2014a
	C	*VMA5*	Decreased	Locked in yeast	Avirulent	Zhang et al., 2017
	D	*VMA8*	–	–	–	
	E	*VMA4*	Decreased	Locked in yeast	Avirulent	Kim et al., 2019
	F	*VMA7*	Decreased	Partial defect	Avirulent	Poltermann et al., 2005
	G	*VMA10*	Decreased	Locked in yeast	Avirulent	Kim et al., 2019
	H	*VMA13*	–	–	–	
V_e_	a	*VPH1*	Decreased	Partial defect	Avirulent	Kane, 2006
		*STV1*	Uchanged	Partial defect	Virulent	Kane, 2006
	c	*VMA3*	Decreased	Locked in yeast	–	Rane et al., 2014b
	c'	*VMA11*	–		–	
	c''	*VMA16*	–		–	
	d	*VMA6*	Decreased	Locked in yeast	Avirulent	Jia et al., 2018b
	e	*C1_10750C_A*				

Studies have shown that the structure of the V-ATPases in vacuoles plays an important role in pH balance and ion homeostasis (Veses et al., 2008). Factors affecting V-ATPase assembly and cellular trafficking also have strong influences on calcium ion homeostasis. All these functions are required for virulence and pathogenesis in *C. albicans*. The deletion of any one of the genes encoding subunits of the V-ATPases creates a Vma deficient (Vma^−^, vacuolar membrane ATPase activity) phenotype. Vma^−^ *S. cerevisiae* and *C. albicans* demonstrate similar functional patterns, showing increased sensitivity to high pH, heavy metal ions, and antifungal drugs (Kane, 2007). *S. cerevisiae* cells with the Vma^−^ phenotype also show slower growth compared to wild-type cells even at pH 5 and have defects in sporulation and germination (Kane, 2006).

The case is similar for *C. albicans*; several studies have established the necessity of V-ATPases subunits in maintaining vacuolar pH and virulence. The *vma4* and *vma10* null mutants of *C. albicans* both show non-acidic compartments and attenuated virulence. Protease secretion is also defective in the null mutants, and this compromises their ability in host cell degradation and in immune evasion (Kim et al., 2019). When *VMA2* expression is repressed, vacuolar acidification is inhibited causing abnormal vacuolar morphology, and autophagy is delayed as visualized by monitoring Ape1-GFP localization. The mutant shows the Vma^−^ growth phenotype and is avirulent in the *C. elegans* infection model (Rane et al., 2014a). The *vma5* and *vma7* null mutants of *C. albicans* are found to have the same defects in vacuolar acidification and are avirulent in a mouse model of systemic candidiasis (Poltermann et al., 2005; Zhang et al., 2017). *C. albicans VMA3* is found to be functionally similar to *S. cerevisiae VMA3* and, when its expression is disrupted, results in the loss of V-ATPase activity and vacuolar acidity. In addition, loss of *VMA3* results in significantly attenuated macrophage killing (Rane et al., 2013). The deletion of Tfp1, the putative *C. albicans* homologue of *S. cerevisiae* Vma1, can cause a defect in vacuolar acidification and strongly reduces virulence (Jia et al., 2014). *VPH2* encodes the homologue of Vma12, which is one of the V-ATPases assembly factors, and *VMA6* encodes subunit d required for V_1_ domain assembly. Disruption of either of these two genes elevates vacuolar pH and weakens the virulence of *C. albicans* (Jia et al., 2018b).

There is a different case for the *VPH1* and *STV1* genes, as they both encode for a subunit of V_0_. Both the *VPH1* and *STV1* genes need to be deleted to show a full Vma^−^ phenotype (Kane, 2006). However, the *vph1* null mutant is unable to acidify vacuolar compartments and is avirulent, while the *stv1* null mutants can have their functions compensated by Vph1 and are shown to be virulent. This study shows that Vph1 plays a more important role in maintaining virulence for *C. albicans* than Stv1, although there is functional redundancy between the two isoforms that makes the effects of losing either one of them less significant than a regular Vma^−^ phenotype (Patenaude et al., 2013).

As well as the genes directly coding for the components of V-ATPase, V-ATPase also require ergosterol to function properly. Erg mutants with disruptions in the last step of ergosterol synthesis also show a Vma^−^ phenotype with an inability to grow in alkaline medium and failure to acidify the vacuole (Zhang and Rao, 2010). This suggests that ergosterol is necessary for V-ATPase activity. Other lipids may play a role in controlling V-ATPase activity as well. Sphingolipids with a C26 acyl group are critical for the activity of V-ATPase (Chung et al., 2003), and deletion of either Sur4 and Fen1, which are critical for sphingolipid biosynthesis, results in a milder version of the Vma^−^ phenotype (Kane, 2006).

The deletion of genes coding vacuolar protein sorting components (VPS) like Vps28 and Vps32 also give rise to similar phenotypes to Vma^−^ with enhanced sensitivity to alkaline pH and weakened virulence (Cornet et al., 2005). The null mutants of a subset of VPS genes like *VPS34* or *VPS15* abolish the uptake of quinacrine into the vacuole and lead to increased sensitivity to high pH with reduced V-ATPase activity due to a vacuolar acidification defect (Sambade et al., 2005). Certain VPS proteins like Vps34 are found to directly interact with Vma7 and may control the assembly of V-ATPase, so the *vps34* null mutant has the same phenotypes as the *vma7* null mutant in terms of vacuolar acidification and lower virulence (Poltermann et al., 2005).

Overall, the Vma^−^ phenotype highlights the vacuole’s role in maintaining ion homeostasis, and morphological transformation can also be impaired when V-ATPases or vacuolar trafficking pathways are defective.

## Hyphal Growth Defects and Cell Wall Changes Through V-ATPases Inactivation

*C. albicans* are more capable of blocking phagosomal maturation and acidification when they have normal filamentation, and *C. albicans* invasion into oral and gastrointestinal tract epithelia involve hyphal form cells (Zhang and Rao, 2010), so filamentation could be used as a trait to assess virulence. The loss of hyphal growth can have several different vacuole-related genetic causes (Chen et al., 2020). *Vma^−^* mutants exhibit different degrees of defects in hyphal development. The *vma3* and *vma7* null mutants have essentially no filamentous growth in liquid Spider medium while filaments can be induced from wild-type cells. The deletion of *TFP1*, *VPH2*, or *VMA6* also gives dramatic attenuation of *C. albicans* filamentous growth (Jia et al., 2014). In addition, the *vph1* mutant shows deficiencies in hyphae formation while the *stv1* null mutant has more normal filamentation. Interestingly, different hyphal development defects correspond with the inability to acidify vacuoles in the *vph1* mutant but to lesser extent in the *stv1* mutant. A link between vacuolar pH and hyphal formation is thus evident, and, as antifungal drugs that disrupt vacuolar pH also block hyphal growth, this suggests the V-ATPases may assist the signaling that induces hyphal formation (Patenaude et al., 2013).

In addition, the decreased activity of V-ATPase may influence cell wall synthesis through a reduction in the transport of secretory vesicles (Marshansky and Futai, 2008). For instance, *vph2* or *vma6* null mutants are hypersensitive to cell wall stresses and their cell wall composition changes significantly; the mutants contain more chitin and less β-1,3-glucan and phosphomannan (Jia et al., 2018b).

## Hyphal Development Defects Caused by Disruption of Vacuolar Trafficking Genes

Hyphal formation in *C. albicans* is not only related to the vacuolar pH regulated by V-ATPases, but also related to vacuolar trafficking. Vacuolar trafficking involves the exchange of substances or vesicles between the vacuole and the endoplasmic reticulum, Golgi, mitochondria and other organelles, and is essential for maintaining the virulence of *C. albicans* (Bianchi et al., 2019). In *S. cerevisiae*, Vps21 was found to mediate vacuolar trafficking *via* an endosomal route, and a *vps21* deletion in *C. albicans* causes a mild reduction in hyphal growth and virulence. Although the null mutant of *aps3* alone does not produce an avirulent strain with a loss of filamentation, loss of function for both *VPS21* and *APS3* shows synthetic effects, generating pseudohyphae without vacuolated compartments and causing a significant decrease in virulence. This suggests that *VPS21* and *APS3* mediate vacuolar trafficking through distinct pathways and that the *APS3* pathway is more significant when endosomal trafficking is disrupted (Palmer, 2010). The *vps34* null mutant also has faulty vacuolar trafficking, with enlarged vacuoles and significantly less hyphal growth (Bruckmann et al., 2000). The *vps11* null mutant has defects in filamentation and secreting proteases, and is completely unable to kill macrophages, resulting in a decrease in virulence (Palmer et al., 2003, 2005). Disruption of *VPS1* by a regulatable tetracycline promoter produces defective filamentation and markedly reduced biofilm formation (Bernardo et al., 2008). It appears that disruption of vacuolar trafficking prevents vacuolation, compromises the regulation of turgor pressure that helps to provide a force for directional hyphal elongation, and prevents necessary factors like V-ATPase subunits from localizing in the vacuole. All these could be reasons why vacuolar trafficking is critical for fungal filamentation (Palmer, 2010).

The *vps11* null mutant has reduced function in secreting proteases and lipases and is completely unable to kill macrophages (Palmer et al., 2003; Veses et al., 2008). Studies also found that *C. albicans* lacking *VPS1* expression, as well as the *vps4* null mutant, have reduced secretion of aspartyl proteases and phospholipase (Bernardo et al., 2008; Lee et al., 2009). The *vps4* mutant causes greatly decreased virulence in both a mouse tail model of disseminated infection and in a *C. elegans* model of infection. The null mutant also has decreased macrophage killing ability and causes less tissue damage to epithelial cells, showing that the pre-vacuolar secretory pathway plays a role in several virulence-related aspects in *C. albicans* (Rane et al., 2014b).

**Figure 1:**
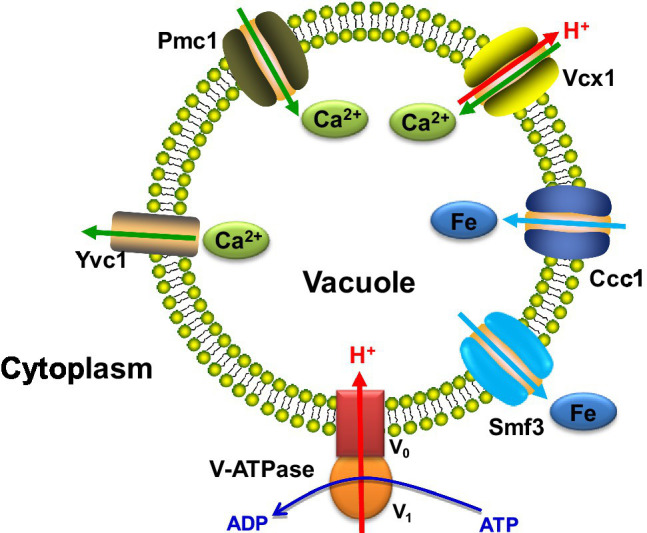
The major ion transporters located on the vacuolar membrane in *C. albicans*. V-ATPase, pumps H^+^ from the cytoplasm to the vacuole. Yvc1, transports Ca^2+^ from the vacuole to the cytoplasm. Pmc1, transports Ca^2+^ from the cytoplasm to the vacuole. Vcx1, the Ca^2+^/H^+^ exchanger. Ccc1, the vacuolar iron importer. Smf3, the vacuolar iron exporter. The arrows represent the direction of ion transport.

In addition to vesicle trafficking, vacuolar calcium and iron transport are crucial for *C. albicans* virulence and hyphal formation. Some proteins involved in vacuolar ion transport are shown in Figure 1. Ca^2+^ participates in various signaling pathways to mediate cellular responses, and a low cytosolic Ca^2+^ concentration is required (Li et al., 2018). The vacuole is the site for calcium storage to maintain the optimum intracellular calcium level. The major vacuolar importer and exporters are the Ca^2+^ channel Yvc1, the Ca^2+^ pump Pmc1 and the Ca^2+^/H^+^ exchanger Vcx1, which are all vital to Ca^2+^ homeostasis (Cunningham, 2011).

Upon a hypotonic shock, vacuolar Yvc1 releases Ca^2+^ into the cytosol. A study on Yvc1’s importance for *C. albicans* shows that the *yvc1* null mutant has a much weaker calcium pulse under alkaline pH or hypertonic shock, and a second fluctuation, where Yvc1 releases vacuolar calcium in response to the stimuli to increase cytosolic calcium levels, is reduced. Yvc1 thus plays a part in mediating the increase of cytoplasmic calcium levels after external stimuli.

In addition, the *yvc1* null mutant shows a reduction in hyphal development, producing mainly pseudohyphae, and has defects in biofilm development and hyphal polarized growth. Yvc1 has a role in activating expression of hypha-specific genes during hyphal growth, and the virulence of *C. albicans* without Yvc1 is highly attenuated in a mouse model of systemic infection. Also, the damage ability of the *yvc1* null mutant is significantly decreased compared to WT during invasion of human epithelial cells.

Yvc1 mediates stress resistance after stimulation by controlling cytoplasmic calcium levels and the subsequent activation of calcium signaling pathways, and it has a role in hyphal growth and re-orientation to host cells. These observations suggest why this putative vacuolar Ca^2+^ channel has an important part in maintaining the virulence of *C. albicans* (Yu et al., 2014).

Pmc1 and Vxc1 sequester Ca^2+^ ions into the vacuole (Cunningham, 2011). The *C. albicans pmc1* null mutant was severely impaired when CaCl_2_ concentrations are high, while the *vxc1* null mutant is unaffected. This suggests a significant role of Pmc1 in calcium homeostasis and stress tolerance. Also, the loss of *PMC1* impairs the cell’s ability to form hyphae, and this negatively affects the *pmc1* null mutant’s biofilm development, which is both related to the high calcium concentration caused by the loss of calcium detoxification performed by Pmc1. Furthermore, the *pmc1* null mutant is avirulent in a mouse model of disseminated infection, while the *vxc1* null mutant shows no difference compared to wild type in these aspects. Pmc1, with its calcium mediation function, has proved to be essential for the pathogenicity and virulence of *C. albicans* (Luna-Tapia et al., 2019).

Iron homeostasis has been found to be critical for the regulation of commensalism and pathogenicity of *C. albicans* (Noble et al., 2017; Tripathi et al., 2020). Because they maintain the major iron pools in fungi, mitochondria and vacuoles play central roles in modulating intracellular iron homeostasis. The vacuolar iron importer Ccc1 and exporter Smf3 are confirmed to regulate both cellular iron levels and hyphal development in *C. albicans*. However, the hyphal development and virulence deficiencies caused by *CCC1* and *SMF3* knockouts are not as significant as those caused by the disruption of the mitochondrial iron transporter *MRS4*. In addition, *CCC1* disruption could rescue the filamentous development and virulence in the *mrs4Δ/Δ* mutant, which suggests an opposing influence of Mrs4 and Ccc1 on iron homeostasis (Xu et al., 2014).

## V-ATPases Subunits and Assembly Factors Maintain Vacuolar Calcium Homeostasis

Vacuolar calcium channels have been found to be affected by other regulators, especially those related to V-ATPases. For example, the absence of the assembly factor Vph2 of the V-ATPase and the loss of Tfp1, the subunit a of the V_1_ domain, causes abnormal localization of Yvc1 and leads to the disruption of calcium transport from the vacuoles to the cytosol (Peng et al., 2020). The *vph2* null mutant has attenuated pathogenicity (Jia et al., 2018b), and the *tfp1* null mutant has significantly increased cytosolic calcium levels, indicating its importance in ion homeostasis (Jia et al., 2015). The *tfp1 pmc1* double mutant has increased disruption in calcium homeostasis compared to the *pmc1* null mutant alone. The *vph2* or *vma6* null mutants give rise to abnormal localization of Tfp1, which consequently affects the vacuolar calcium channel Yvc1 (Jia et al., 2018a). Overall, the proteins involved in vacuolar protein or ion transport mentioned above are critical for maintaining the pathogenicity of *C. albicans*.

## Conclusion and Perspectives

In summary, studies have found *C. albicans* virulence is affected by several aspects of vacuolar function including vacuolar pH, vacuolar trafficking, calcium homeostasis, and iron homeostasis. The master pump V-ATPase maintains vacuolar pH and is crucial for pathogenesis and virulence, and its loss of activity also affects hyphal growth and calcium channel function. As well, the calcium and iron channels are necessary for filamentation and biofilm development in *C. albicans*. Vacuolar trafficking also controls vacuolar morphology, V-ATPase activity, autophagy, and hyphal growth, elaborating the role of *VPS* genes in the pathogenicity of *C. albicans*. This vacuolar trafficking process also involves the interaction of multiple protein families, such as Rho/Rab GTPases, guanylate exchange factors, the HOPS (homotypic fusion and vacuole protein sorting) complex, and the SNARE (soluble NSF attachment protein receptor) complex; this extensive system has not been detailed in this focused review (Bröcker et al., 2010). Moreover, vacuolar fusion can influence hyphal compartments; the highly fragmented vacuoles in *C. albicans* enable hyphal extensions and septation with reduced branching frequencies. These interconnected pathways may have further potential as targets for future antifungal drug discovery. Thus, further research is still needed to fully understand both morphogenesis and the role of vacuoles in the mechanisms behind pathogenesis and virulence in *C. albicans*.

## Author Contributions

LY conceived and wrote the review. QL and YJ conceived and searched the references. All authors contributed to the article and approved the submitted version.

## Funding

This review is funded by the National Natural Science Foundation of China (82173867), Shanghai Science and Technology Innovation Action Plan, International Science and Technology Cooperation Project (21430713000), Shanghai Science and Technology Support Project in the Field of Biomedicine Project (19431901300), Shanghai Sailing Program (19YF1458800) and the key project of the National Natural Science Foundation of China (81830106), and Shanghai Pujiang Program (21PJD081).

## Conflict of Interest

The authors declare that the research was conducted in the absence of any commercial or financial relationships that could be construed as a potential conflict of interest.

## Publisher’s Note

All claims expressed in this article are solely those of the authors and do not necessarily represent those of their affiliated organizations, or those of the publisher, the editors and the reviewers. Any product that may be evaluated in this article, or claim that may be made by its manufacturer, is not guaranteed or endorsed by the publisher.
